# A Social Media Study of Portrayals of Bipolar Disorders on YouTube: Content and Thematic Analyses

**DOI:** 10.2196/67129

**Published:** 2025-04-25

**Authors:** Eric Mayor, Lucas M Bietti

**Affiliations:** 1 Division of Clinical Psychology and Epidemiology University of Basel Basel Switzerland; 2 Department of Psychology Norwegian University of Science and Technology Trondheim Norway; 3 Department of Innovation WSB University Warsaw Poland

**Keywords:** bipolar disorder, YouTube, social media, content analysis, thematic analysis

## Abstract

**Background:**

Individuals with mental disorders frequently use YouTube to express themselves, reach an audience, or as a means of understanding their condition. Testimonies posted on YouTube provide longer and richer perspectives than the short posts found on other social media platforms. Research focusing on the depiction of mental disorders on YouTube is blossoming. Bipolar disorders (BDs) are disabling mood disorders. The diagnosis of any mental disorder, and more so BD, is often a life-changing event. However, no published study has investigated the portrayal of diagnoses of BD on YouTube.

**Objective:**

This study aims to investigate the portrayals of BDs on YouTube, focusing on the diagnosis narratives and their accompanying narrative context, in particular, reports of personal experiences and reactions.

**Methods:**

We performed a manual content analysis of 39 testimonies (women: n=24, 62%) depicting BDs and their diagnosis by individuals with BD. We also performed a thematic analysis of the corpus relying upon a deductive and inductive approach.

**Results:**

Our manual content analysis revealed that portrayals included the disclosure of diagnoses of BD-I (as per both coders’ agreement: 10 testimonies) and BD-II (11 testimonies) to a similar extent. The reactions to the diagnosis were mostly negative (8 testimonies), followed by positive (5 testimonies), while fewer portrayals indicated a denial of the condition (4 testimonies). Several portrayals made mention of issues in the areas of money and accommodation (15 testimonies), profession and education (13 testimonies), and relationships (20 testimonies). Medication (31 testimonies) and psychotherapy (23 testimonies) were often mentioned as part of treatment for BD, most generally in positive terms. The 8 themes emerging from the thematic analysis were: “reactions on diagnosis, treatment, and health care professionals’ expertise,” “trial and error in medication,” “positive effects of BD,” “disability, stigma, and shame,” “loss,” “family planning and genetics,” “identity change (psychological and physical),” and “human social relationships.”

**Conclusions:**

Overall, our results underline the complexity and richness of the depiction of the diagnosis of BD and its narrative context, and highlight the importance of the moment of the diagnosis, medication, and psychotherapy. Our study emphasizes the need for further exploration of the impact of social media on mental health awareness.

## Introduction

### Background

Bipolar disorders (BDs) are disabling mood disorders and are associated with 12-14 years earlier mortality [1,2]. In the United States, the annual direct health care costs of individuals with a BD are on average 86% higher than those of the general population [3]. The median age of onset for BDs is 21 years old (with important variability) [4]. Thus, in the majority of cases, individuals with BD are affected by the disorder and its potentially devastating consequences for the entirety of their adult lifetime. Despite their relatively low prevalence (1% to 2%) [2,5], BDs induce a considerable burden on afflicted individuals and society.

Individuals affected with symptoms of the disorder might engage in online information searching to cope with their disturbing nature [6]. Young individuals facing mental health issues often search for information online, as this affords them anonymity which helps them avoid being stigmatized [7]. Social media in particular are a frequently used source of information, with YouTube being the most relied upon of such sources [8]. Sharing experiences online is also considered a means of better understanding oneself and one’s condition and is used by individuals with the purpose of educating others and reducing mental health stigma [9].

A burgeoning field of research is interested in the depiction of mental disorders on YouTube [10-14]. However, there is currently a lack of research examining the information conveyed in relation to the diagnosis of a BD. Here, we examine the depiction of BD in testimonies of individuals diagnosed with the disorder relying upon manual content analysis. We also perform a thematic analysis of the corpus relying on an inductive and deductive approach.

### Perspectives on Mental Disorders on YouTube

Analyses of data from social media platforms relying upon short texts and images (eg, Twitter [subsequently rebranded X], Facebook, and Instagram) typically require very large sample sizes. They allow for studying general patterns in affect trajectories at the population level [15-17] and the unobtrusive estimation of mental disorder symptomatology at individual and regional levels [18-20]. The examination of longer-form testimonies on YouTube allows for a more rich and detailed understanding of the portrayal of mental disorders on the internet. Unlike other social media platforms such as Twitter, Facebook, and Instagram [18-21], the scientific interest in the depiction of well-being and mental health on YouTube has only recently increased.

Past studies have investigated the portrayal of mental disorders on YouTube. Some have examined general aspects of mental disorders, while others have focused on specific health conditions. We briefly summarize important aspects of such studies. Devendorf et al [11] notably examined how videos on depression depicted causes, consequences, curability, portrayal perspectives, disorder timeline, and the strengths and identity of individuals with depression. The authors highlighted that biopsychosocial portrayals gain more views while biological portrayals might reduce blame but also increase perceptions of danger and lower recovery expectations. Gaus et al [12] investigated personal accounts of depression in popular videos and their comments. The majority of videos (73%) mentioned youths, suicidality, or self-harm and discussed treatment options, but only a minority advocated for clinical treatment. Approximately 5% of comments mentioned clinical treatment, while only 2% advocated for it. References to suicide or self-harm appeared in 7.3% of comments. Oliphant [22] examined user engagement with YouTube videos on depression, BD, and mental health in general. In self-posted videos (the majority) users recounted personal experiences, while other categories of video posters included commercial entities (most likes on average), organizations or government (most comments on average for mental health–focused videos), television or media (most comments on average for BD videos), and universities (most comments on average for depression-focused videos).

Sangeorzan et al [9] identified 3 major themes in videos of individuals identifying with serious mental illnesses: “Minimizing Isolation and Reducing Feelings of Loneliness Through Vlogging,” “Vlogging as Therapy,” and “Fighting Stigma.” The authors highlighted the potential of sharing experiences with serious mental illness to foster self-understanding and reduce stigma. Choi et al [23] found that most videos on college mental health depicted the perspective of students, followed by domain experts. They found that individual posters primarily shared experiential knowledge, while other sources (eg, media, universities) emphasized information sharing and promoting help-seeking. McLellan et al [24] identified 5 major themes in randomly sampled comments on popular YouTube videos related to mental health stigma: “Community Building,” “Personal Experiences of Mental Illness,” “Personal Experiences of Stigma,” “Debates of Mental Illness.” and “Explanations for Mental Illness.” Authors observed that while the biomedical model may reduce stigma by offering medical explanations, it often neglects psychosocial factors.

MacLean et al [14] found that most portrayals of generalized anxiety disorder (GAD) in their sample were personal accounts shared by individuals, which in the majority included mentions of symptoms (eg, worry, panic) and treatment options (eg, support groups, medication, and cognitive behavioral therapy). The etiology of GAD was addressed in approximately one-fourth of the videos. Examining YouTube videos on agoraphobia, Erdogan Kaya and Erdogan Akturk [13] found that 50% provided general information, 28% shared lived experiences, and 22% focused on overcoming anxiety. Videos by professionals were rated as higher in informational quality than those by nonprofessionals, which often received more views and likes. Abhishek et al [25] examined the accuracy of content in the depiction of obsessive-compulsive disorder on YouTube. They found that only a minority contained misleading information about compulsions and obsessions. Few videos included information on the biological basis of obsessive-compulsive disorder or pharmacological treatments, while psychotherapy was mentioned in most. These aspects were not associated with engagement. The portrayal of trichotillomania was examined by Ghate et al [26]. Most videos featured self-filmed narratives, followed by advertisement videos and documentaries. A large proportion of videos featured mentions of hair pulling, the negative consequences associated with the disorder, and management strategies. Strategies for hiding hair loss were also presented. Most videos were created by White individuals, which may have limited representation and relevance for racial minorities. Suresh et al [27] analyzed the content of YouTube videos on anorexia nervosa. Most videos provided information on symptoms, treatment, rehabilitation, and etiology, while 42% featured personal accounts from patients. While content quality did not differ across sources (eg, physicians, news media, patients, and health care organizations), over 70% were made by posters without health care qualifications which may raise accuracy concerns.

Woloshyn and Savage [28] examined how individuals who self-identify with borderline personality disorder (BPD) depicted the condition on YouTube. They assessed both user characteristics and video content through content analysis. Most videos were monologues aiming to foster an understanding of the user’s experience and educate viewers about the disorder. The most commonly reported symptoms included fear of abandonment, unstable relationships, feelings of emptiness, and identity disturbance. Self-disclosure might increase self-esteem and promote awareness but may also risk reinforcing negative self-perceptions. King and McCashin [29] performed a thematic analysis of comments to videos of four YouTube channels about BPD, leading to five themes: (1) sharing advice, support, and encouragement; (2) vlogs destigmatizing, informing, and educating; (3) solidarity, relatability, and personal connection; (4) intense, unstable intrapersonal, and interpersonal functioning; and (5) prompting disclosures about mental health struggles. Vlogs provided peer support and helped combat internalized stigma.

Athanasopoulou et al [10] explored attitudes toward schizophrenia in Finnish- and Greek-language videos and found most depicted individuals with schizophrenia negatively. The videos often included stigmatizing beliefs, such as personal responsibility for the disorder and fears of contagion. However, some videos also highlighted more positive aspects, such as recovery potential, empowerment, and solidarity. Although some videos claimed to be educational, their accuracy was questionable. The study found no significant differences between Finnish- and Greek-language videos. Interested in the accuracy of schizophrenia portrayals on YouTube, Nour et al [30] found that the most commonly depicted symptoms were negative symptoms, persecutory delusions, mood disturbances, disordered thoughts, and auditory-verbal hallucinations. However, about a third of the videos depicted symptoms of other disorders or included no schizophrenia symptoms and about a third were ambiguous in their diagnostic portrayal. These findings suggest that schizophrenia representations on YouTube are inconsistent, with some videos providing accurate depictions while others contribute to misinformation.

Finally, Bakombo et al [31] examined comments on YouTube videos on autism spectrum disorder using thematic analysis. The majority of videos were educational, with 30% discussing personal experiences and 12% focusing on daily life. Anecdotes appeared in 40% of comments, addressing behavioral manifestations, public perceptions, and self-concept. Expressions of personal feelings were balanced in valence. Stereotypes, as well as discussions on etiology, behavior, or interventions, were mentioned in only a few videos. Commenters also reacted to the videos, expressing agreement or disagreement and providing additional explanations.

The studies mentioned above with their significant focus on the accuracy of information presented in the videos and engagement provide a valuable ground for the understanding of the depiction of mental disorders and viewership. While other research has examined individuals self-identifying with a mental condition or the perspective of others (eg, mental health professionals), to our knowledge, no study so far specifically investigated the disclosure of a diagnosis of BD and its accompanying narrative context. The diagnosis of BD in particular is often a life-changing moment because it brings clarity to years of emotional turbulence, explaining extreme mood swings and behaviors. Or because it may reduce the breadth of potential accomplishments individuals may perceive as reachable, because of internalized stigma or the reality of living with the disorder. Often, it allows individuals to access appropriate treatments, such as medications and psychotherapy, that can significantly improve their quality of life. Investigating self-disclosed diagnosis, rather than self-identification with a disorder, is also important because it allows examining the reactions to the diagnosis, including lack of agreement with it and whether individuals think another diagnosis would more aptly describe their difficulties.

### The Study

Here, we examined 39 YouTube testimonies which included the experience of a diagnosis of a BD. The aim of the study was to investigate the portrayal of a BD diagnosis on YouTube, focusing on personal experiences and reactions. To do so, we examined these portrayals by (1) systematically coding and quantifying relevant aspects such as the diagnosis of BD-I or BD-II, the types of reactions to the diagnosis, functional issues, and treatment options depicted in the testimonies and (2) exploring themes such as individuals’ reactions to treatment, as well as the broader impact of BDs on various life domains, including relationships, identity, and the experience of stigma.

We used testimony-level manual content analysis to quantify the presence of various relevant aspects in the testimonies. Content analysis is “the systematic, objective, quantitative analysis of message characteristics” [32]. There are different forms of content analysis, for instance, whether the content analysis is performed manually or automatically; whether the categories are derived theoretically (top down) or empirically (bottom up) [33,34]. Additionally, emergent coding “is somewhere between a purely empirically derived model and a purely theoretical one” [33]. Emergent coding affords a rigorous examination of selected aspects of a corpus, guided both by the literature and the specificity of the documents under examination. Such an approach is common in qualitative research and is also represented in the field [12].

On the other hand, we identified major themes in the testimonies through thematic analysis [35,36]. Thematic analysis is an atheoretical and flexible qualitative method used to identify, analyze, and interpret patterns of meaning (themes) within a corpus [36]. This analysis provides a deeper understanding of how BD influences lived experiences, particularly around diagnosis and ongoing management of the disorder. Understanding lived experiences permits discovering how people with BDs perceive and handle their difficulties. These testimonies offer valuable insights into the emotional, social, and existential aspects of the disorder. Through the testimonies we examined, thematic analysis allowed us here to better understand the reality of living with a diagnosis of BD.

## Methods

### Sample

We used the YouTube application programming interface (API) version 3 to search for the videos at the end of May 2024. We used the search string “diagnosed, bipolar” for 2 different 12-month periods. The YouTube API requires indicating a maximal number of results to be retrieved. We chose to limit to 500 results for each 12-month period because although we were expecting fewer than 500 matching videos per 12-month period, we wanted to be certain not to miss any. We were interested in individual testimonies of people with BD (made in the location of their choice) who posted on their own channel and discussed their diagnosis. We performed the screening of the videos in June 2024 (the content analysis and thematic analysis followed until August 2024). We included the first published eligible video for each speaker over the considered total period. Only videos with a duration of more than 1 minute and a transcription available on YouTube were included. We excluded videos that were not in English (videos with less than minimal switching between languages were also excluded) or that were not intelligible. In order to maintain a degree of similarity between the testimonies, that is to create a corpus of comparable verbal material, we also excluded videos that included interactions with other people (unless minimal exchanges, like: “Hi!,” “Hi!”), that included introductions by others, or when the speaker engaged in extensive description of their environment. After assessment of the testimonies based on channel name, title, and description and watching the videos when relevant (based upon what precedes), 39 testimonies were included in the corpus. In other words, the number of documents was determined by the results of the search and screening process.

### Ethical Considerations

We did not seek ethical approval for this study. This social media study did not involve participant recruitment. The sample consisted of self-posted YouTube testimonies. As part of their user agreement with YouTube, users grant permission for the use of their content as enabled by the platform. Additionally, prior research in the field typically did not report ethical approval [23]. Video identifiers and channel names were replaced by placeholders (eg, Testimony1; Channel1). No channel names or identifiers are provided in this manuscript or its supporting materials. Only paraphrased excerpts are included as examples.

### Engagement Statistics and Video Metadata

We used the YouTube API version 3 to download engagement statistics (eg, number of views, number of likes, and number of comments) and the metadata of the videos (eg, date published and duration).

### Transcripts

Automatic speech recognition is automatically performed on videos uploaded on YouTube: Machine learning algorithms convert the speech into text when the video matches an available language. The quality of transcripts may be affected by background noise, accents, or complex language [37,38]. After the speech is converted into text, transcripts are synchronized with the video’s timeline to match the speech. YouTube allows users to manually edit the transcripts or delete them.

The transcripts of the eligible videos were obtained directly from YouTube: they were copy-pasted and manually checked by us. Mentions of individuals’ names were replaced considering the relationship to the speaker (eg, HusbandFirstName and TherapistName), the names of medications were replaced by MedicationName, and locations and institutions were also replaced (eg, CityName and UniversityName). We removed from the transcripts the introductions to the videos that were clearly indicated as such (eg, titled “Intro”), as introductions are frequently reused across many videos published on the same channel.

### Content Analysis

For our content analysis of the corpus, we relied upon a form of emergent coding [33]. Such an approach has been relied upon previously by social media researchers interested in mental health [34]. As experienced researchers interested in well-being in social media, we developed the categories that follow (Table 1) based on the literature and the testimonies and improved the categories based on the discussion of individual testimonies. In other words, these categories were not based upon preexisting typologies but were conceived by us with regard to the aims of the study, existing literature about mental disorders, and their relevance to the corpus. Not only do these categories inform upon the experiences of individuals with BDs, but they also highlight what information is made available to viewers as personal accounts of experience about mental disorders can influence the beliefs of others [27,28,39].

**Table 1:** Categories of manual coding of testimonies portraying BD^a^ in YouTube videos.

Type	Category	Example (rephrased)	κ	Percent agreement
Diagnosis	Has BD-1 diagnosis	“I was diagnosed with bipolar disorder type 1”	0.71	87.18
Diagnosis	Has BD-2 diagnosis	“I am Bipolar 2”	0.77	89.74
Diagnosis	Positive reaction to the diagnosis	“I am so glad I was diagnosed early”	0.65	89.74
Diagnosis	Negative reaction to the diagnosis	“I was devastated”	0.67	87.18
Diagnosis	Denial of the diagnosis	“I do not have bipolar disorder” [despite mention of receiving the diagnosis]	0.77	94.87
Issues	Money and accommodation issues	“I gave away all my belongings”	0.79	89.74
Issues	Professional and educational issues	“The manager fired me”	0.58	79.49
Issues	Relational issues	“I berated my friends”	0.24	64.10
Treatment	Mention of psychotherapy	“My psychotherapist told me …”	0.59	82.05
Treatment	Mention of medication	“You have to take your meds”	0.83	94.87
Treatment	Mention of side effects of medication	“There are a lot of side effects”	0.43	74.36
Other	Seeing good in BD	“I can do so much during hypomanic episodes”	0.45	76.92
Other	Mention of stigma or the need to break stigma	“There is stigma to bipolar disorder”, “It’s not okay to be stigmatized”	0.49	82.05
Other	Mention of uncertainty toward the future	“I don’t know what will happen to me”	0.04	71.79

^a^BD: bipolar disorder.

The categories are as follows.

- Has BD-1 or BD-2 Diagnosis: Due to the higher duration and frequency of manic episodes in BD-1 compared with hypomanic episodes in BD-2, the diagnosis of BD-1 has starker implications for a person’s prospects of an ordinary life than a diagnosis of BD-2 [2].
- Issues money and accommodation, professional and educational, and relational: A diagnosis of BD (more so for BD-I) is frequently associated with different functional deficits. The *DSM-5-TR* (*Diagnostic and Statistical Manual of Mental Disorders* [5th Edition, Text Revision]) [40] mentions “(…) marked impairment in social or occupational functioning (…)” in criteria C for a manic episode, whereas criteria C for a hypomanic episode [40] requires “(…) unequivocal change in functioning that is uncharacteristic of the individual when not symptomatic (…).” We coded mentions of issues that preceded, were concomitant, or followed the diagnosis in the narration of events. Simple mentions without mentioned chronology were also coded.
- Positive, negative, or denial reaction to the diagnosis: The reaction to the diagnosis can also have important implications, notably in relation to treatment compliance [41]. The literature frequently mentions that denial is a frequent reaction from individuals with BD [42,43].
- Mention of psychotherapy, medication, or side effects of medication: Appropriate care for any mental disorder can be decisive in its progression; this is even more true for BDs [44,45]. Experiencing important side effects is not uncommon among individuals with prescribed antipsychotics and mood stabilizers (category with insufficient interrater agreement) [46]. This can lead to less compliance with treatment [2].
- Seeing good in BD: The enjoyment of the disorder, particularly hypomanic episodes and their symptoms is not infrequent and is another factor affecting compliance with treatment [47].
- Mention of stigma or the need to break stigma: Stigma can be related to reluctance to seek care and lack of compliance with treatment, among others [48,49].
- Uncertainty toward the future: Beliefs toward the disorder (eg, unpredictability of depressive or manic episodes) can have implications with regard to treatment adherence [50]. The uncertainty associated with the course of bipolar disorder is an important challenge for patients, their therapists, and their families [51,52].

Both authors coded the testimonies for the presence or absence of the categories based on the transcripts and verification using the videos, while the sex (female or male) of the speaker (100% agreement) was coded using the videos alone. The interrater agreement in the coding of the testimonies is presented in Table 1, along with examples for the categories. It can be noted that while most categories featured a very good or appropriate Cohen κ, it was insufficient for some categories most strikingly (“mention of uncertainty”), which reflected the more conservative coding of one of the coders. While reporting the Cohen κ as a measure of interrater agreement is a common practice when performing content analysis [33], some authors in the field [10,12,14,25] reported the percentage of agreement instead of or in addition to Cohen κ. In our study, the percentage of agreement between coders ranged from 64.1% (relational issues) to 94.87% (denial of diagnosis and mention of medication). The thematic analysis pursues goals that are complementary to the content analysis.

### Data Analysis

#### Frequencies of Content Categories and Engagement

We present descriptive statistics for the frequencies of content categories and duration and engagement variables.

#### Thematic Analysis

Thematic analysis offers in-depth engagement with a corpus and allows flexibility in the discovery of patterns of meaning within the examined data [36].

While content analysis offers the advantage of quantification, thematic analysis complements it by enabling an in-depth exploration of textual data to uncover patterns of meaning within the corpus. Our approach to the thematic analysis was as follows. We started taking notes on potential themes during the manual coding of the testimonies. After the coding was done, we listed themes of interest and examined the transcriptions in relation to the video-level coding again in light of these themes, sometimes also watching the videos. We updated the themes. This was followed by coding of the themes in the transcripts including the extraction for each theme of relevant textual material in each transcript, if any. At the moment of describing the themes based on the collated excerpts, the themes were sometimes merged, as there were important overlaps in excerpted material (eg, initial themes “reactions on diagnosis and medication” and “professional expertise” were merged into the theme “reactions on diagnosis, treatment, and health care professionals’ expertise,” as a critique of expertise was grounded upon the reactions on the diagnosis and medication and at times only implicitly communicated). The thematic analysis thus entailed reexamining and revising the themes on several occasions and was performed both deductively and inductively [36]. In the description of the themes below, we present the final result of our analysis with examples that were paraphrased for ethical reasons (with changes to dates and values). Paraphrasing the examples allows protecting the identity of the authors of the testimonies and is becoming a common practice in social media research focusing on mental health [29,39,53].

## Results

### Video Durations and Engagement

Table 2 presents the duration of the videos as well as engagement metrics (number of views, likes, and comments). As can be noticed from the dispersion of these values, there was ample variation in the data.

**Table 2:** Durations and social engagement with testimonies portraying bipolar disorder in YouTube videos.

	Mean (SD)	Total (thousands)
Duration (seconds)	755.59 (486.25)	29.5
Views	1140.00 (5176.08)	44.5
Likes	61.29 (246.76)	2.3
Comments	13.18 (27.36)	0.501

### Content Analysis

**Figure 1:**
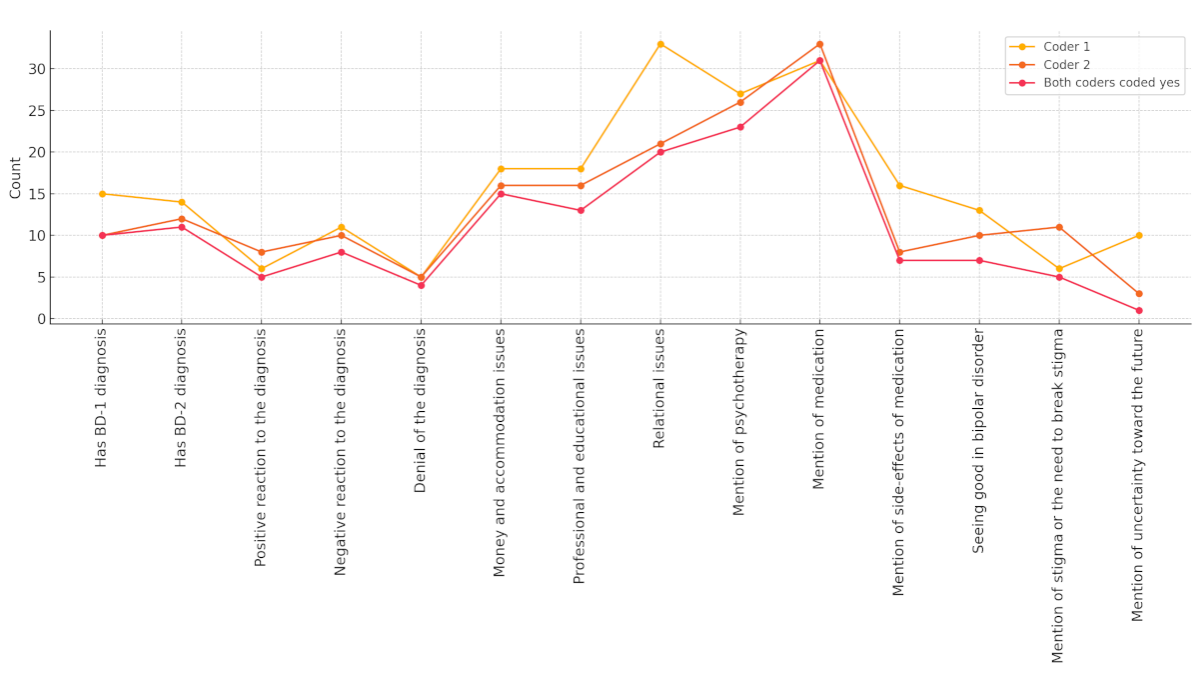
Frequencies of coded categories in the testimonies portraying bipolar disorder in YouTube videos. BD: bipolar disorder; Coder 1: frequency of testimonies coded yes by coder 1; Coder 2: frequency of testimonies coded yes by coder 2; Both coders coded yes: Frequency of testimonies coded yes by both coders 1 and 2.

The manual coding of the corpus was performed at the testimony level in order to provide an overview of the content of the testimonies both based upon elements of relevance in the literature and care of patients with BD and the examination of the testimonies. The frequencies of the coded categories are provided in Figure 1. Our comments are based here on the frequencies for “Both coders coded yes,” which mainly mirrors the more conservative ratings of coder 2*.* In most instances, though, these comments are valid for the ratings of both coders.

About the same number of testimonies were proposed by individuals revealing a diagnosis of BD-1 compared with BD-2. More testimonies indicated a negative reaction to the diagnosis, than a positive reaction. Yet, a denial of the diagnosis was the least frequently rated reaction to the diagnosis. The area of issues mentioned most frequently in the videos were relational issues (category with poor interrater agreement), followed by issues in relation to money and accommodation as well as work or education. Most testimonies made mention of both medication and psychotherapy. The majority of the testimonies did not mention the side effects of medication. A nontrivial minority of testimonies were rated as indicative of a BD enjoyment (category with insufficient interrater agreement). Again, a nontrivial minority of the testimonies indicated stigma was an issue in relation to mental disorders overall, or to BD specifically (category with insufficient interrater agreement).

### Thematic Analysis

#### Reactions on Diagnosis, Treatment, and Health Care Professionals’ Expertise

##### Diagnosis

Being diagnosed with BD often came as a shock. Testimonies often mentioned the news was devastating and overwhelming (and other negative emotions; A1, Table 3). Denial was also a reaction to the diagnosis (A2), while others immediately endorsed the diagnosis (A3). For many, adaptation to the diagnosis took a very long time (sometimes lasting for years). A few videos made mention of not knowing what to expect following their diagnosis (A4). The question of having children or not following a diagnosis of BD was mentioned a few times (see “Family Planning and Genetics subsection for more details), with different opinions on the matter.

**Table 3:** List of examples of paraphrased excerpts from the testimonies portraying bipolar disorder in YouTube videos^a^.

Example ID	Text
A1	“I’m relieved to have a diagnosis so I can get effective treatment”
A2	“We disagree about my diagnosis”
A3	“I am at peace with that diagnosis”
A4	“What can I look forward in my life?”
A5	“The mix of medications has been life-saving”
A6	“I didn’t want to take the medication initially”
A7	“Mood stabilizers are a blessing”
A8	“That medication made me manic”
A9	“I felt awful taking the meds”
A10	“Forgetting to take the drugs a few times sent me to the hospital”
A11	“I didn’t feel like myself with this medication”
A12	“If the symptoms don’t fit bipolar, it is not bipolar”
A13	“I had the incorrect diagnosis for many years”
A14	“I am a strong supporter of psychotherapy”
A15	“Psychotherapy just doesn’t work for me”
B1	“Medication might not work after some time, so you have to change”
B2	“The medication I am taking is not effective”
B3	“My mental health decreased when my dose was decreased”
B4	“I became a better parent with this medication”
B5	“[the medications] aren’t currently effective”
C1	“You might be seen as an overachiever”
C2	“I experience highly creative and energetic nights”
C3	“You see yourself as invulnerable”
C4	“I am happy with the life I have and wouldn’t change anything”
D1	“My disability doesn’t allow me to work”
D2	“The situation is embarrassing and I am ashamed”
D3	“When I mention that I’m bipolar people react as if I were crazy”
D4	“I felt deeply guilty over my behavior during my manic episode after it ended”
E1	“I just lost it all”
E2	“I was about to spend more money than I could afford on loved ones”
E3	“At that time, I couldn’t see my buddies”
E4	“I would have graduated and achieved what I wanted in life”
E5	“I couldn’t feel like myself anymore”
F1	“There is a history of bipolar disorder in my family”
F2	“She explained what she was doing to stay good mentally”
G1	“I have difficulty understanding who I have become”
G2	“Who I was before physically is no longer who I am”
H1	“I wouldn’t have succeeded without my friends”
H2	“I often feel drained after talking with people”
H3	“My father and my mother struggled with addiction”
H4	“My childhood was marked by chaos because I was bullied at school”

^a^Theme A: Reactions on diagnosis, treatment and health care professionals’ expertise; Theme B: Trial and error in medication; Theme C: Positive effects of bipolar; Theme D: Disability, stigma and shame; Theme E: Loss; Theme F: Family planning and genetics; Theme G: Identity change (psychological and physical); and Theme H: Human social relationships.

##### Medication

Some testimonies indicated that being diagnosed with BD was a life-saving event, allowing for the prescription of adequate treatment (A5). Initial reluctance toward medication was not uncommon (A6), yet many highlighted their satisfaction with their current medication (A7)—at times mentioning the treatment they received to alleviate depressive symptomatology triggered their first manic episode (A8). Antipsychotics and mood stabilizers were at times linked to a variety of side effects or simply were not considered effective (A9). The importance of compliance with medication was frequently highlighted (A10). But some abandoned their treatment abruptly, either because of side effects or because of the enjoyment of manic or hypomanic symptoms that were precluded by the medication. Some were apprehensive regarding whether medication could alter their identity (A11).

##### Clinicians’ Expertise

The expertise of clinicians was sometimes questioned, either by individuals who still did not agree with the diagnosis or because of the side effects of medication received for major depressive disorder (A12). Some individuals also questioned the tardiness of their diagnosis, delaying proper treatment (A13). Psychotherapy was also considered helpful by many (A14). A few individuals found psychotherapy unhelpful and shared their frustration that it did not work for them (A15).

#### Trial and Error in Medication

Testimonies provided a detailed account of individuals’ challenging experiences with managing mental health through medication. They described how the process often involved trial and error, with the wrong medications sometimes worsening their condition, leading to further adjustments and reevaluations (B1).

Despite these efforts, some individuals still struggle with BD symptoms, indicating the ongoing difficulty of finding effective treatment (B2). One testimony also touches on a specific period when medication was reduced, which led to severe difficulties (B3). Some testimonies mention that medication, while not perfect, can improve daily functioning (B4). Individuals also expressed frustration over the need to constantly adjust medications due to side effects or diminishing effectiveness over time (B5).

Despite the difficulties, the individuals acknowledged the necessity of medication to manage their condition, even though it remains an ongoing, challenging process. The testimonies also briefly mentioned experiences with various medications, including mood stabilizers, antidepressants, and antipsychotics, and undergoing electroconvulsive therapy. However, none of these treatments provided a definitive solution, highlighting the complexity of managing mental illness.

#### Positive Effects of BD

The testimonies sometimes provided detailed and personal reflections on the positive effects of BD, particularly focusing on the experiences of mania and hypomania. These descriptions highlighted the heightened emotions, creativity, and energy that accompany manic and hypomanic episodes (C1). During these periods, a sense of euphoria was often conveyed. The testimonies illustrated how mania and hypomania led to extreme energy and creativity (C2).

Testimonies also referenced the sense of grandiosity that accompanied mania and hypomania, where some individuals believed they were destined for greatness. This inflated self-perception contributed to an overall feeling of being on top of the world (C3). At times, they also expressed contentment with the diagnosis and a sense of identity shaped by BD, with individuals finding joy in the unique experiences it brought. They were not running from their condition but instead embracing it as a part of their identity, which has shaped their lives in meaningful ways (C4).

#### Disability, Stigma, and Shame

Testimonies include descriptions related to disability, stigma, and shame. Individuals reported that living with BD often results in financial difficulties, with some relying on Social Security Disability Insurance (SSDI; D1). While SSDI provides essential support, it can also feel degrading, contributing to feelings of inadequacy and shame. The limited income from SSDI, combined with concerns about losing vital health insurance if they attempt to work, traps many in a cycle of dependency (D2). Individuals often experience intense stigma and shame due to societal attitudes. They felt ashamed of not being “normal” and perceived that their condition was treated more harshly than other mental disorders (eg, depression or anxiety). This stigma can lead to social avoidance of the topic and a lack of understanding from others, reinforcing feelings of isolation and marginalization (D3). Shame was often associated with guilt over past behaviors during manic or hypomanic episodes, such as impulsive actions or hurtful remarks, which can lead to the loss of friendships and strained relationships (D4). Disability, stigma, and shame were three closely intertwined topics in the testimonies and were also associated with the theme of “loss.”

#### Loss

The theme of loss was present in some of the testimonies. In some cases, the loss occurred prior to the diagnosis (eg, loss of a relative, loss of a job or housing; E1); in other instances, the loss of wealth appeared to be constitutive of BD symptomatology (eg, giving away belongings, buying things one cannot afford, losing friends over objectionable or exuberant behavior, quitting jobs multiple times for little reasons; E2); and finally, loss was also presented as a consequence of the diagnosis or disorder (eg, loss of social standing, being ostracized because of diagnosis, being fired because working in some sectors with a BD diagnosis is against company policy or legal obligations of the employer, loss of social competencies; E3). The loss of opportunities was also at times mentioned in relation to tardive diagnosis (E4). The loss of self and one’s identity were also occasionally mentioned (E5).

#### Family Planning and Genetics

Testimonies focused on the role that genetics (eg, BD running in families) plays in being diagnosed with BD and how this diagnosis affects decisions regarding family planning (eg, being bipolar and pregnant). These testimonies discussed the challenges and considerations involved. Several individuals mentioned how BD has been present in their families (F1). However, having a close relative with BD was also described as a source of support and reliable information. Sharing experiences and information about coping with BD and its symptoms within families was seen as positive, especially when family members were diagnosed earlier and could transmit their experiences to newly diagnosed individuals (F2).

The testimonies acknowledged that BD is, to a significant extent, determined by genetics. However, they also emphasized that epigenetics, including environmental factors and lifestyle, plays a role as well. Regarding family planning, individuals mentioned their plans to monitor their children’s mental health closely and seek early intervention if necessary. Overall, the notion that being bipolar should prevent one from having children was largely rejected. Instead, the focus was on preparing to support their children’s potential mental health needs.

#### Identity Change (Psychological and Physical)

Testimonies described the psychological and physical changes experienced by individuals with BD, reflecting on identity, self-perception, and the impact of medication. This theme conveys a complex and often painful journey of navigating life with BD, marked by a constant battle between maintaining identity and managing the effects of the disorder and its treatment. The diagnosis of BD was a pivotal moment, providing a framework for understanding their experiences.

Some individuals expressed confusion about their identity (G1), feeling like different people merged into one. They struggled with the notion of whether they were truly themselves or a product of their disorder.

The physical toll of the disorder and its treatment was significant, with individuals reporting weight gain, loss of energy, and a decline in overall health (G2). These changes were intertwined with the emotional and psychological challenges of living with bipolar disorder, including anxiety, obsessive thoughts, and the fear of future episodes.

#### Human Social Relationships

Testimonies reflected on how human social relationships affected the diagnosis of BD in both positive and negative ways. The role of friends and family is central to these testimonies. They emphasize the importance of surrounding themselves with supportive and positive individuals (H1), as opposed to those who drain their energy (H2). Family history is another significant topic present in the testimonies. Several individuals described a conflicted childhood, including parental addiction (H3) and bullying (H4).

Maintaining healthy relationships proved at times challenging. Family members, partners, and friends often encouraged individuals with BD to seek professional help, leading to a diagnosis of BD. These testimonies highlighted the necessity of having a reliable support system and being kind to oneself and others. This support was crucial in understanding and managing their condition.

## Discussion

### Principal Findings

Personal accounts of mental health issues on YouTube are an important aspect of online mental health discussions [22]. Past studies have investigated the portrayal of mental disorders on YouTube. To our knowledge, no study so far specifically investigated the disclosure of a diagnosis of BD and its accompanying narrative context. We presented the first study investigating the portrayal on YouTube of a received diagnosis of a BD and its accompanying narrative context.

In our corpus, there were no major differences in the frequency of testimonies disclosing a diagnosis of BD-1 or BD-2, aligning with existing epidemiological estimates [5]. Reactions to the diagnosis were mostly negative, followed by positive, while fewer individuals indicated a denial of the condition. The testimonies frequently mentioned psychotherapy and medication as treatment options which were generally presented positively, while some testimonies discussed side effects. Some testimonies emphasized the importance of treatment compliance, while others revealed noncompliance, with individuals discontinuing treatment due to side effects or even deliberately triggering manic episodes to enjoy the symptoms. This could relate in part with the identification with the disorder for some individuals.

The testimonies frequently mentioned issues pertaining to the financial (and accommodation), professional and educational areas, and even more so relational areas. Guilt was portrayed as an emotion arising from the recollection of how the speakers had acted in past interactions. Sometimes these relationships come to an end because of such behavior. Loss was also portrayed in several testimonies in relation to other objects, such as wealth, opportunities (not having graduated from college), or competencies (not being capable of interacting with friends anymore). Relationships were also mentioned in relation to having a family member to talk with about BD and its management. Other sources of support were mentioned by other individuals, highlighting the importance of social relationships in the management of BD. Social relationships were also mentioned at times as a source of stress, or even as playing a role in the origin of the disorder (eg, childhood mistreatment). A few testimonies highlighted a feeling of being different individuals merged into one. Stigma was at times mentioned by the speakers (eg, mention of discrimination or differences in treatment), including self-stigma (eg, the shame of not being capable of offering a contribution to society).

The reaction to receiving the diagnosis varied qualitatively between mentions of being devastated to mentions of being relieved because the diagnosis allowed proper treatment. The reaction was more often negative than positive or in denial. Denial is frequently observed in the clinical context with patients diagnosed with BD [42,43]. Some individuals in the testimonies expressed disagreement with their diagnosis, either at the time it was given or even at the time of sharing their account. Descriptions of denial and other reactions to a diagnosis are rare in previous research on the depiction of mental health disorders on YouTube, which typically examined self-identification with a mental disorder. Research has shown that comments on YouTube videos about BPD often included personal disclosures, such as viewers expressing gratitude to the video creators for helping them better cope with a recent diagnosis [24,29]. Additionally, comments frequently offered advice and shared experiences from the perspective of individuals diagnosed with BPD, serving as efforts to challenge and reduce mental health stigma.

Medication and psychotherapy are a central part of the treatment of BDs [1,2,54]. Such findings align with a study on the portrayal of depression on YouTube [12]. Several papers on YouTube videos focusing on mental disorders examined the question of treatment in their corpus. For instance, MacLean et al [14] and Abhishek et al [25] noted that 60% or more of the videos they examined mentioned a form of treatment. Past research [22,23] has highlighted the explicit promotion of help-seeking in YouTube videos. This aspect was also occasionally present in our corpus. Side effects of medication for BD are common and vary depending on the pharmacotherapy, with frequent issues including weight gain, metabolic dysregulation, sedation or drowsiness, and akathisia [55]. Other studies on different disorders have also highlighted discussions of treatment side effects in YouTube videos [11,24], with skepticism toward the biomedical model frequently appearing in mental health testimonies on the platform [24]. The difficulty of finding the correct combination and dosage of medication was also highlighted in the management of BD. The avoidance of medication in some cases might have the function of preserving identity rather than simply a hedonic function [56]. Such a finding is in line with the literature which suggests that an important proportion of individuals with BDs appreciate certain aspects of the condition (eg, enhanced mood, creativity, and energy during manic or hypomanic episodes) complicating treatment [2,56-58]. A study found that a quarter of bipolar patients do not want the disorder removed even if it could be [56] and as many as 60% of patients with BD are not compliant with medical treatment, especially during manic episodes [2].

Psychotherapy might be more discussed in YouTube videos about BD than other disorders (eg, GAD [14]; trichotillomania [26]). Psychotherapy was most often considered important for the treatment of BD, while a few revealed dissatisfaction and discouragement. Some testimonies highlighted some degree of identification with the disorder, while others mentioned not being the same person anymore, either physically (eg, important weight gain) or psychologically (not recognizing themselves in whom they had become).

A difference is that past studies of YouTube videos focusing on mental health did not report on guilt and loss which are frequently considered central aspects of depressive episodes [59,60], Yet, such aspects were often mentioned in the testimonies we examined, at times, in relation with relational or professional issues, and issues with money and accommodation.

Family history of BD and genetic components play an important role in the etiology of BDs [61,62]. The heritability of BDs lies around 70% [63]. Considerations on this aspect have been identified in YouTube videos in previous studies [14]. YouTube testimonies examined in other studies have also highlighted the importance of receiving (and providing) support as well as being understood for individuals with mental disorders [9,23,26,27,29]. The question of relationships, and more generally participation in society, extends to the issue of stigma. Individuals with BD often face stigma from others and may even endorse such beliefs themselves (self-stigma and internalized stigma) [49,64]. Stigma affects not only the individuals but also their families, potentially leading to ostracism from family members [48,49]. Individuals with mental disorders might not seek professional help for fear of mental health stigma [65]. Addressing mental health stigma on YouTube is an important goal of personal testimonies [24]. This was frequently described in previous studies in the field [23,28,29,31].

### Implications

This study provided essential elements to the understanding of the portrayal of BDs on social media like YouTube. Our results have shown that the specific challenges faced by individuals and the sometimes-related question of the relationships as central to the lived experience with BD. Overall, the knowledge of information available to potential viewers might allow communication campaigns to be more targeted toward providing information elements that are lacking from the testimonies. Another approach might be, depending upon the strategic goals of such campaigns, to highlight elements similar to those in the testimonies to enhance their credibility. Similarly, clinicians might be helped in their care for affected individuals by knowing more about the information available on social media. Such knowledge is also of interest to researchers, who might be interested in exploring the distinctions in the portrayals of different disorders. We have already highlighted above some of the differences.

### Limitations and Further Studies

The study has several limitations. It relied on a relatively small sample of 39 testimonies. Individuals who shared their BD diagnosis on YouTube may have been more willing to publicly disclose personal experiences, potentially resulting in an overrepresentation of certain viewpoints (eg, those more comfortable discussing their diagnosis online). The data were self-reported, which may introduce biases due to factors like memory inaccuracies, self-presentation strategies, or reluctance to disclose negative experiences [66,67]. The study focused solely on English-language testimonies, excluding non-English-speaking individuals, which may overlook the experiences of diverse cultural groups who might conceptualize and cope with BD differently [68-70]. These limitations could prevent capturing the full diversity of experiences among individuals diagnosed with BD. YouTube’s engagement dynamics (eg, likes, comments, views) may have influenced how individuals presented their experiences, encouraging content that is more engaging or meaningful for others [22].

Future research should explore how perceptions of BD evolve over time, focusing on coping strategies and treatment adherence through video testimonies. It is important to examine cross-cultural and linguistic differences in portrayals of mental disorders, as well as the role such portrayals play in stigma reduction. Analyzing how YouTube's algorithms influence the visibility of BD-related content could offer valuable insights into the unique challenges individuals with BD face when sharing their testimonies on social media. Furthermore, the potentially differentiated portrayal of BD and other disorders depending on developmental periods and social media platforms (eg, TikTok) should be examined.

### Conclusions

In this study, we have examined the portrayal of the personal experience of individuals diagnosed with BD. Our results notably highlighted the breadth of the issues reported by the individuals and the importance of treatment. Our study emphasizes the need for further exploration of the impact of social media on mental health awareness. Such research can provide valuable insights into the emotional, social, and existential dimensions of BD, contributing to the development of more tailored health care interventions.

## Abbreviations

API: application programming interface
BD: bipolar disorder
BPD: borderline personality disorder
*DSM-5-TR*: *Diagnostic and Statistical Manual of Mental Disorders* (Fifth Edition, Text Revision)
GAD: generalized anxiety disorder
SSDI: Social Security Disability Insurance

## Data Availability

Data is deposited on the OSF Registries (coding of the testimonies; osf.io/4kqa9).
